# CD14 genotype and functional dichotomy of CD14+ and CD14- cells are associated with activated immune response and development of Chagas dilated cardiomyopathy

**DOI:** 10.1590/0074-02760200110

**Published:** 2020-10-28

**Authors:** Germano Carneiro Costa, Manoel Otávio da Costa Rocha, Paulo Eduardo Alencar de Souza, Diego Felipe SA Melo, Paula Rocha Moreira, Kenneth John Gollob, Maria do Carmo Pereira Nunes, Walderez Ornelas Dutra

**Affiliations:** 1 Universidade Federal de Viçosa, Florestal, MG, Brasil; 2 Universidade Federal de Minas Gerais, Faculdade de Medicina, Programa de Pós-Graduação em Infectologia e Medicina Tropical, Belo Horizonte, MG, Brasil; 3 Pontifícia Universidade Católica de Minas Gerais, Faculdade de Odontologia, Belo Horizonte, MG, Brasil; 4 Universidade Federal de Minas Gerais, Departamento de Morfologia, Laboratório de Biologia das Interações Celulares, Belo Horizonte, MG, Brasil; 5 AC Camargo Cancer Center, São Paulo, SP, Brasil; 6 Instituto Nacional de Ciência e Tecnologia de Doenças Tropicais, Salvador, BA, Brasil

**Keywords:** Chagas cardiomyopathy, CD14, polymorphism

## Abstract

We aimed to investigate the association of *CD14* -260C/T (rs2569190) polymorphism and Chagas cardiomyopathy and the functional characteristics of CD14+ and CD14- monocytes upon infection with *Trypanosoma cruzi*. We observed an association between the T- genotype (absence of allele -260T) related to low CD14 expression and the dilated cardiomyopathy type of Chagas disease. Furthermore, we observed that CD14- monocytes showed a more activated profile upon *in vitro* infection with *T. cruzi* than CD14+ monocytes. Our findings suggest that T- genotype is associated with susceptibility to develop Chagas dilated cardiomyopathy, likely linked to the *T. cruzi*-induced inflammatory profile of CD14- monocytes.

Chagas disease cardiomyopathy is the most severe outcome of the infection with the protozoan *Trypanosoma cruzi*, responsible for over 10,000 deaths annually. There are currently over 100 million people at risk of infection with *T. cruzi* and, of those, at least 30% may develop the deadly cardiomyopathy. There is no vaccine to prevent the disease and no markers to identify individuals at risk of developing the severe form. 

The great clinical variability of chronic Chagas disease is directly associated with the host’s immune response.^1^ ^,^ ^2^ Research from our group has previously shown that CD14+ monocytes are critical cells in determining the inflammatory and anti-inflammatory immunological profile observed in chronic cardiac and asymptomatic (indeterminate) Chagas patients, respectively.^1^ In addition, *T. cruzi* strains have been associated with severe and mild infections and profoundly affect the response of CD14+ monocytes, inducing an inflammatory or regulatory profile in these cells, respectively.^3^

CD14 is a leucin-rich receptor, mainly expressed on the surface of innate cells, especially monocyte/macrophages. CD14 can also occur in a soluble form (sCD14), which can act a receptor on cells that do not express CD14 on its surface, such as dendritic cells.^4^ CD14 expression is influenced by a functional polymorphism rs2569190 (-260 C/T) in the promoter region of the CD14 gene.^5^ The polymorphic variant (T) has been associated with higher transcriptional activity of the receptor and high expression levels of sCD14 and this polymorphism has been associated with susceptibility to several inflammatory and infectious diseases.^6^

Although CD14 lacks an intracellular domain, which prevents signalling, it plays a fundamental role on innate response as it function as a co-receptor for toll-like receptors (TLRs). It has been demonstrated that GPI-mucins derived from trypomastigote forms of *T. cruzi* stimulate host cells via TLR2 in association with CD14 and via TLR-4.^7^ ^,^ ^8^ Thus, CD14 might be important in defining human monocyte response to *T. cruzi*. 

Our working hypothesis was that CD14 functional polymorphism is associated with the clinical outcomes of human Chagas disease. Thus, we sought to investigate if there is an association between rs2569190 polymorphism-derived genotypes and the susceptibility to develop the most severe form of Chagas disease. Our results showed an association of the T- genotype (absence of allele -260 T) and the severe dilated clinical form of Chagas disease. In addition, using an *in vitro* system of *T. cruzi* infection, we observed that the parasite induces distinct responses in human CD14+ and CD14- monocyte subpopulations, which might be associated with the role of these cells during Chagas disease progression.

Chagas’ disease patients were under medical responsibility of Dr Manoel O. Rocha and his group and were recruited at the Centre for Training and Reference in Infectious and Parasitic Diseases Ambulatory (CTR-DIP) from the Medical Center of Federal University of Minas Gerais, Brazil. This cross-sectional study was performed with patients from endemic areas in the state of Minas Gerais, Brazil. The intervention group was composed by 185 Chagas patients and 63 non-Chagas individuals (N), as determined by negative specific serological tests, participants in the control group. All Chagas patients were in the chronic phase of infection with well-defined clinical forms. Classification based on clinical, radiological and echocardiographic criteria led to the stratification of patients into three groups: indeterminate patients (IND) (n = 64), asymptomatic, positive specific serology for *T. cruzi*, normal electrocardiogram, normal cardiac and digestive radiological evaluation (some patients presented minimal alterations on echocardiogram and were also considered as asymptomatic); non-dilated cardiopathy patients (C) (n = 53), with positive specific serology, alterations in electrocardiogram such as right/left branch block and different degrees of functional alterations in the conduction system, no heart dilation; dilated cardiomyopathy patients (DC) (n = 68), with positive specific serology, alterations in clinical, radiological and, especially, echocardiographic exams, showing severe cardiomyopathy with heart enlargement. Uninfected individuals (N) were used to ascertain that no major changes in genotype distribution occurred in Chagas patients (data not shown). Exclusion criteria were diabetes *mellitus*, thyroid dysfunction, renal insufficiency, chronic obstructive pulmonary disease and rheumatic or autoimmune diseases. The demographic characteristics of clinical groups are summarised in Table.

**TABLE:** Demographic characteristics and genotype and allele frequency of the *CD14* -260 C/T polymorphisms in Chagas disease patients

	Clinical groups		
	Indeterminate (n = 64)	Non-dilated cardiac (n = 53)	Dilated cardiac (n = 68)
Gender			
Male	29 (45.0)	22 (41.5)	41 (60.0)
Female	35 (55.0)	31 (58.5)	27 (40.0)
Age range			
Range	18-73	29-67	22-73
Mean ± SD	43.3±10.4	46.6±9.4	50.86±11.2
LVEF (%)	65.6±5.05	62.5±5.6	43.35±11.86
Genotype			
CC	17 (26.5)	21 (40.0)	29 (43.0)
CT	34 (53.0)	25 (47.0)	28 (41.0)
TT	13 (20.5)	7 (13.0)	11 (16.0)
T^*-a*^	17 (26.5)	21 (39.6)	29 (42.6)
T^*+a*^	47 (73.5)	32 (60.4)	39 (57.4)
Allele			
C	68 (53.0)	67 (63.0)	86 (63.0)
T	60 (47.0)	39 (37.0)	50 (37.0)

Data is shown as number (%) of subjects. χ^2^ likelihood test were used for 3 × 2 contingency table comparison among different clinical groups, degrees of freedom (df) = 2. Fisher exact test were used for 2 × 2 contingency table (comparison used for allele T carriage and allele frequency). *a*: dilated cardiac (DC) × indeterminate (IND): χ^2^ = 3.79, p = 0.05; odds ratio: 2.05 (95%CI 0.97-4.28); LVEF: *left ventricular ejection fraction; SD: standard deviation.*

For genotyping analysis, cells were obtained through oral swab performed with a sterile plastic spatula. DNA extraction was performed using the silica method, as previously done.^9^ *CD14* rs2569190 polymorphism was assessed by RFLP. PCR primers sequences were 5’-CTAAGGCACTGAGGATCATCC-3’ and 5’-CATGGTCGATAAGTCTTCCG-3’ with expected PCR product size of 418 bp as previously described.^6^ Briefly, PCR was carried out in a total volume of 25 μl, with 55ºC used for annealing temperature. PCR products were digested with 5 units of HaeIII enzymes at 37ºC for 4 h. Digestion products of 263 bp + 155 bp were obtained for C allele, while T allele remained with 418 bp length band. Genotype was diagnosed with silver-stained 10% acrylamide gel. For CD14 expression, blood samples were collected from six healthy individuals (N) by puncture of the peripheral vein into Vacuntainer tubes containing heparin. Adherent-cells preparations were obtained from PBMC purified cells.^2^ To determine infectivity and functional characteristics of CD14+ and CD14- subpopulations, adherent cells were exposed to CFSE-labelled Y strain trypomastigotes of *T. cruzi,* using 10 trypomastigotes/cell and analysed by flow cytometry.^1^ We then determined the percentage and intensity of CFSE+ cells in CD14+ and CD14- subpopulations. Functional characterisation of CD14+ and CD14- monocytes was performed by measuring surface expression of CD80 and CD86 and intracellular cytokines TNF-alpha and IL-10. A minimum of 40,000, 20,000 and 30,000 events on monocyte gate were counted for infectivity, surface marker expression and cytokines analysis, respectively. Analyses were done using FlowJo software.

Statistical analysis was performed using the JMP statistical software (SAS, Cary, USA). Chi-square test was used to assess association between genotype/allele frequency and study groups. Odds ratio (OR) with 95% confidence interval were performed for 2 × 2 contingency table. The study groups followed Hardy-Weinberg equilibrium, comparing the expected with the observed genotype frequencies. Wilcoxon paired test was applied for comparisons of means from distinct treatments, within the same group. Statistically significant results were considered when p ≤ 0.05.

All individuals included in this work were volunteers and procedures were in accordance with ethical standards and with the Helsinki Declaration of 1975, revised in 1983. Written informed consent was obtained from all subjects after ethical approval by the National Committee (CAAE: 49211015.0.0000.5153) and complete medical care was offered independent of their agreement to participate in this study.

Frequencies of genotypes and allele variants of rs2569190 polymorphism are shown on Table. Neither genotype nor allele frequency were associated with clinical forms of Chagas disease. However, when we categorised genotypes by polymorphic allele -260T carriage (-260CT and -260TT) as T+ group, with potential high CD14 expression, and T- group (-260CC), associated with low CD14 expression, we observed that the T- genotype was associated with DC, when compared with IND (p = 0.05; OR: 2.05; 95%CI 0.97-4.28). Interestingly, this association was not seen between IND and C.

To further characterise CD14+ and CD14- subpopulations, we evaluated infectivity, expression of surface molecules CD80 and CD86 and cytokines (TNF-alpha and IL-10) by cells from healthy donors upon *in vitro* after exposure to *T. cruzi*. Infectivity was expressed as CFSE expression in CD14+ and CD14- cells. The percentage and intensity of CFSE+ cells were greater in the CD14+ subpopulation as compared to CD14- monocytes (Figure A). CD80 expression was upregulated after *in vitro* exposure to *T. cruzi* in both cell subpopulations (Figure B). However, CD86 expression was only upregulated in CD14+ cells after exposure to *T. cruzi* (Figure B). CD14- showed higher levels of CD80 and CD86 receptors than CD14+ population in non-stimulated cultures (Figure B). This was maintained after *T. cruzi* exposure for CD86, but not CD80 expression, emphasising the upregulation of CD80 by the parasite (Figure B). 

**Figure:**
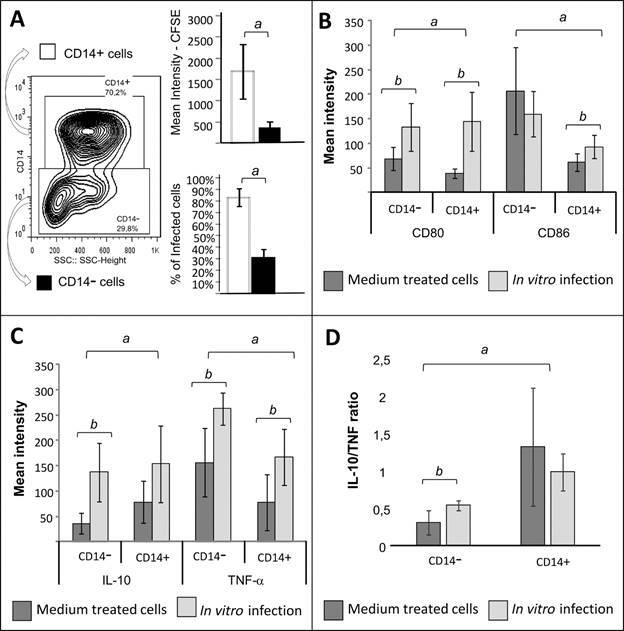
Determination of infectivity and functional analysis of CD14+ and CD14- cells. (A) Density plot shows the selection of CD14+ and CD14- cells using side-scatter vs CD14 expression plot, in which the expression of CFSE was evaluated. Results are expressed as the mean intensity of CFSE expression and percentage of CFSE+ cells. (B) Analysis of CD80 and CD86 mean intensity of expression by CD14+ and CD14- cells non-stimulated (medium treated cells) and after *in vitro* infection with *T. cruzi* trypomastigotes. Results are presented as mean ± standard deviation (SD). (C) Analysis of IL-10 and TNF-alpha mean intensity of expression by non-stimulated CD14+ and CD14- cells (medium treated cells) and after *in vitro* infection with *T. cruzi* trypomastigotes. Results are presented as mean ± SD. (D) Analysis of IL-10/TNF ratio in CD14- and CD14+ cells. Statistically significant results were obtained** **using the Wilcoxon paired test (p **<** 0.05) for comparisons of different cell populations or different treatment groups.

Analysis of IL-10 and TNF-alpha expression showed that both cytokines were upregulated after infection in CD14+ and CD14- cells (Figure C). Interestingly, while TNF-alpha expression was higher in CD14- monocytes, IL-10 expression was higher in CD14+ cells (Figure C). We determined the IL-10/TNF ratio to further evaluate the balance between modulatory or inflammatory response in CD14+ and CD14- subpopulations. This analysis showed that CD14- cells exhibit lower ratio than CD14+ before and after exposure to *T. cruzi*, further confirming the inflammatory bias of the CD14- cells (Figure D).

Identifying genetic risk factors for parasitic infections could provide important leads for improved therapies and vaccines.^10^ Studies have suggested that the host’s genetic background seems to play an important role in the complex interaction with parasite, affecting the clinical course of Chagas disease.^9^ ^,^ ^11^ ^,^ ^12^ Here we demonstrated that the genotypic variant T- of *CD14* polymorphism rs2569190 is associated with DC. This genotype was higher in cardiac patients displaying the most severe dilated form as compared to indeterminate individuals, but not in the cardiac patients with less severe cardiomyopathy. We also observed that CD14- and CD14+ subpopulations exhibit distinct functional profile, especially in response to *T. cruzi* infection. While CD14+ cells were preferably infected by parasites and displayed a modulatory phenotype, CD14- cells were significantly less infected and exhibited more activated profile, with higher levels of CD86 and TNF-alpha. 

The polymorphic allele -260T was associated with high expression of surface CD14 and sCD14. Conversely, the lack of this allele (T- genotype) is associated with low expression of CD14.^6^ A functional study of this polymorphism, carried out in heart disease patients, showed that six deceased patients and 17 out of 20 carried at least one T-allele, revealing a linear association between the event frequency and genotype, which suggests a dominant effect of the T-allele.^5^ This result does not conflict with our findings, since Chagas cardiomyopathy is caused by a parasitic infection and the aforementioned study involved idiopathic cardiopathy. Moreover, Guerra et al.^13^ showed an association of a haplotype profile with -260T allele, along with other two SNPs in CD14 promoter gene in linkage disequilibrium, with higher plasma levels of sCD14. Therefore, association findings for -260T marker could be related to other functional variant in linkage disequilibrium. Nevertheless, allele -260T on CD14 promoter might be a tag-SNP for cardiomyopathy severity in Chagas disease. Low expression of CD14 may impact the efficiency in recognising and responding to pathogens, as CD14 represents a major receptor related to first activation response of cells from the monocytic-lineage. A polymorphism located in the *TIRAP* gene, an important player of innate immunity signal transduction, and another in TLR4, were also associated with Chagas disease outcome.^12^ This data reinforces the idea that gene polymorphisms associated with innate responses may be good predictive markers for susceptibility to severe forms of Chagas disease.

We further evaluated the functional profile of two subpopulations of monocytes defined by the expression of CD14 (CD14+ and CD14-) using cells from healthy individuals. Previous studies have suggested that the loss of CD14 may represent the rising of distinct cell phenotype, with distinct functional properties, which could be macrophage like (CD14+/MHC-II+) and/or myeloid dendritic cells (CD14-/MHC-II+) migrating to tissues.^14^ Our data showed that CD14+ and CD14- cells indeed display distinct characteristics upon first contact with *T. cruzi*. First, CD14+ cells displayed higher infection by *T. cruzi* trypomastigotes than CD14- cells. Moreover, CD80 and CD86 were distinctively expressed in CD14+ and CD14- cells in response to *T. cruzi*. CD80 and CD86 have distinct kinetics of surface expression and, while they bind to the same ligands on T cells surface, CD80 displays higher activity for CTLA-4, which sends modulatory signals to T cells, while CD86 binds preferably to CD28, important for T cell activation.^15^ Here we showed an upregulation of CD80 expression by the parasite in both cell populations. Previous work by our research group showed that indeterminate patients display higher levels of CTLA-4 expression in their CD8 T cells.^2^ This, together with the upregulation of CD80 induced by the parasite in monocytic cells, may reflect an attempt to dampen T-cell activation. Interestingly, while CD86 was not upregulated by the parasite in CD14- cells, its expression was already higher in this population, suggesting better ability to activate T cells as compared to CD14+ cells. 

Our results showed that parasite induces expression of IL-10 and TNF-alpha in both subpopulations. However, the induction of TNF-alpha was even higher in CD14- cells. Moreover, CD14+ cells exhibited a modulatory profile, with higher IL-10/TNF-alpha ratio, while CD14- cells exhibited a lower ratio. Therefore, CD14- and CD14+ cells represent two subpopulations with distinct functional potential. 

Overall, our findings showed that T- genotype, related to polymorphism rs2569190 in *CD14* gene, is associated with the dilated cardiac form of Chagas disease, but not with the non-dilated form, and may arise as a marker of susceptibility to disease severity. In addition, it is clear that the differential expression of CD14, which is influenced by gene polymorphism, defines functionally distinct populations, which behave differently upon *T. cruzi* challenge, with the CD14- cells displaying activated and more inflammatory profile. Thus, differential CD14 expression may have an important influence in the development of Chagas cardiomyopathy.
